# Inhibition of hypothalamic MCT1 expression increases food intake and alters orexigenic and anorexigenic neuropeptide expression

**DOI:** 10.1038/srep33606

**Published:** 2016-09-28

**Authors:** Roberto Elizondo-Vega, Christian Cortés-Campos, María José Barahona, Claudio Carril, Patricio Ordenes, Magdiel Salgado, Karina Oyarce, María de los Angeles García-Robles

**Affiliations:** 1 Departamento de Biología Celular, Facultad de Ciencias Biológicas, Universidad de Concepción, Concepción, Chile; 2 Whitehead Institute for Biomedical Research, Cambridge, Massachusets, 02142, USA; 3 Facultad de Medicina, Universidad San Sebastián, Concepción, Chile

## Abstract

Hypothalamic glucosensing, which involves the detection of glucose concentration changes by brain cells and subsequent release of orexigenic or anorexigenic neuropeptides, is a crucial process that regulates feeding behavior. Arcuate nucleus (AN) neurons are classically thought to be responsible for hypothalamic glucosensing through a direct sensing mechanism; however, recent data has shown a metabolic interaction between tanycytes and AN neurons through lactate that may also be contributing to this process. Monocarboxylate transporter 1 (MCT1) is the main isoform expressed by tanycytes, which could facilitate lactate release to hypothalamic AN neurons. We hypothesize that MCT1 inhibition could alter the metabolic coupling between tanycytes and AN neurons, altering feeding behavior. To test this, we inhibited MCT1 expression using adenovirus-mediated transfection of a shRNA into the third ventricle, transducing ependymal wall cells and tanycytes. Neuropeptide expression and feeding behavior were measured in MCT1-inhibited animals after intracerebroventricular glucose administration following a fasting period. Results showed a loss in glucose regulation of orexigenic neuropeptides and an abnormal expression of anorexigenic neuropeptides in response to fasting. This was accompanied by an increase in food intake and in body weight gain. Taken together, these results indicate that MCT1 expression in tanycytes plays a role in feeding behavior regulation.

The hypothalamus controls feeding behavior and glucose homeostasis through the integration of diverse peripheral signals, such as leptin, insulin, ghrelin and glucose. There is great interest in understanding the molecular and cellular mechanisms that control the detection and response to glucose, known as hypothalamic glucosensing, given its putative contribution to the development of metabolic diseases, including diabetes and obesity. It has been demonstrated that both neurons and glial cells of the basal hypothalamus are able to respond to glucose concentration^1^,^2^,^3^. Thus, different groups support the idea that hypothalamic glucosensing is an example of glia-neuron coupling mediated by specialized ependymal cells, tanycytes.

Tanycytes are highly polarized glial cells lining the lower side and the floor of the third ventricle (3V); they can be classified as α1, α2, β1 and β2, according to their ventricular wall distribution and their neuronal nuclei contacts^4^,^5^. The arcuate nucleus (AN), master regulator of nutritional and neuronal signals^6^,^7^ is in contact with α2-tanycyte processes^1^,^4^,^5^ as well as β1-tanycytes through their basal processes, which project at the junction between the AN and median eminence (ME)^8^. Both *in vitro* and *in situ* analyses have shown that tanycytes respond to glucose by increasing intracellular free Ca^2+^ levels, a result of ATP production via glycolysis^1^,^2^. Tanycytes also release lactate through the monocarboxylate transporters, MCT1 and MCT4^8^.

MCT1 is located in the short cellular processes of ventral β1-tanycytes, which are in close contact with neurons that mainly release the orexigenic neuropeptides, Agouti-related protein also called Agouti-related peptide (AgRP) and neuropeptide Y (NPY). In contrast, MCT4 is located in the long cellular processes of dorsal β1-tanycytes, which are in close contact with neurons that release mainly the anorexigenic neuropeptides, pro-opiomelanocortin (POMC) and cocaine- and amphetamine-regulated transcript (CART)^8^. Immunohistochemistry studies have shown high levels of MCT2 in the membrane of AN neurons that is not detected in glial cells. MCT2 is involved in the monocarboxylate influx in both anorexigenic and orexigenic neurons, suggesting that monocarboxylates could regulate the activity of these two neuronal types^9^. Recently, immunohistochemical analysis has revealed MCT1 expression in neurons that express NPY^10^. However, electrophysiological AN NPY neurons activity measurements did not reveal any direct modulation in response to hydroxybutyrate^10^.

Current evidence shows that neuroendocrine neurons can detect changes in glucose concentrations through direct or indirect mechanisms^11^,^12^,^13^,^14^,^15^,^16^,^17^. We propose that these neurons sense glucose changes, at least in part, by an indirect process mediated by MCTs, tanycytes and lactate. In response to an increase in glucose concentration, tanycytes would release lactate, which would act as an intercellular messenger of the metabolic state of the organism, informing and regulating the activity of AN neurons. To test this hypothesis, we generated an adenoviral vector that inhibits the expression of MCT1 (AdshMCT1), which principally transduces tanycytes when is injected in the basal 3V. We evaluated changes in the expression of orexigenic and anorexigenic neuropeptides in response to intracerebroventricular (icv) glucose injection, and also determined feeding behavior changes during the fasting-feeding transition.

Due to the high prevalence of obesity worldwide, there has been an enormous effort to understand how nutrients, hormones and neuropeptides can modulate eating responses, which has led to the development of new ways to study eating behavior with animal models. In this context, a distinction has been made in how satiation, defined as mechanisms causing meal termination, and satiety, defined as mechanisms causing delay in the initiation of a new meal, can be measured^18^,^19^. For this reason, we have analyzed the effect of MCT1 inhibition on meal pattern parameters, such as meal frequency, intermeal intervals, meal size and meal duration. Our results show that MCT1 inhibition in tanycytes dysregulates neuropeptide expression and alters hunger and satiety signals, which impact eating behavior. These results support the involvement of glia-neuron interaction in hypothalamic glucosensing.

## Results

### Adenoviral MCT1 inhibition in tanycyte cultures

**Figure 1:**
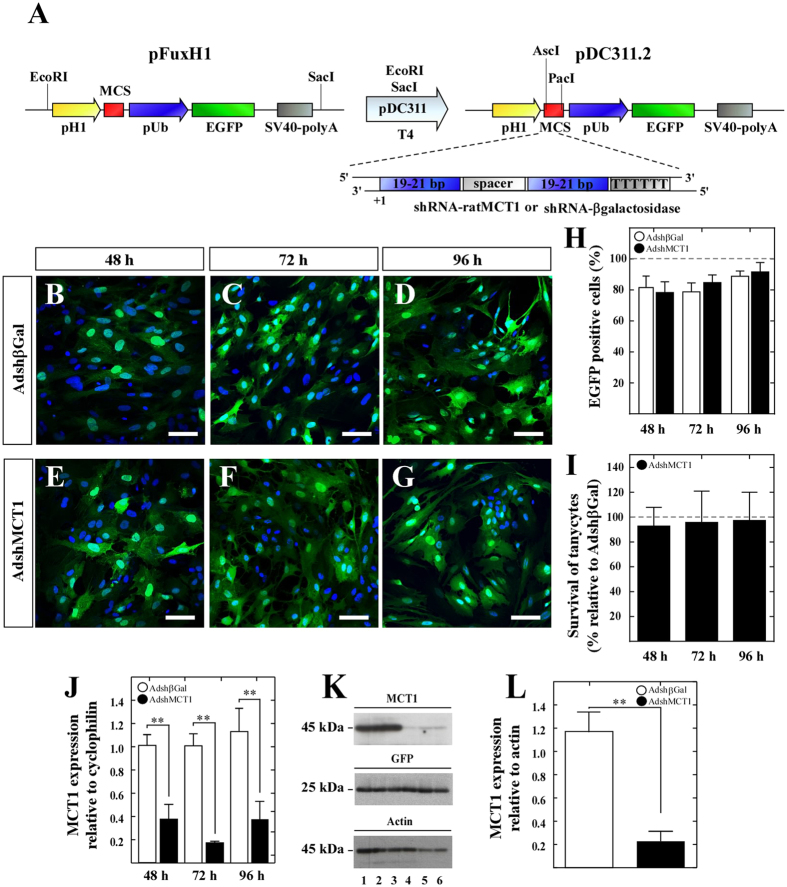
Knockdown of MCT1 in primary cultures of hypothalamic tanycytes. (**A**) Experimental protocol that shows the construction of adenoviral shuttle vector. The cassette encoding the H1-promotor, multicloning site (MCS), ubiquitin promoter, EGFP and SV40 polyA contained in the Fux vector was cloned into the PDC311 adenoviral shuttle expression vector for generating the vector, PDC311.2. The shRNAs were cloned into the MCS. **(B–G)** Temporal EGFP expression in tanycytes cultures transduced for 48, 72 and 96 h with AdshβGal (**B–D**) or AdshMCT1 (**E–G**). Nuclei were stained with TOPRO-3 (blue). (**H)** Quantification of EGFP expression normalized to total cells in the tanycyte cultures transduced with AdshβGal (open bars) or AdshMCT1 (closed bars) at 48, 72 and 96 h. (**I)** Quantification of survival of tanycytes cultures transduced with AdshMCT1 at 48, 72 and 96 h relative to survival of cells transduced with AdshβGal. (**J)** Q-RT-PCR analysis of MCT1 in tanycytes infected with AdshβGal (open bars) or AdshMCT1 (closed bars) at 48, 72 and 96 h. (**K)** Western blot analysis of MCT1, GFP and actin. Lanes 1–3: Total cell extracts treated for 96 h with AdshβGal; lanes 4–6: total cell extracts treated for 96 h with AdshMCT1. (**L**) Semi-quantitative densitometric analysis of MCT1 protein expression relative to actin. GFP: Transduction control. Actin: Loading control. **p < 0.01 (unpaired t-test). Scale bar: 25 μm. MCS, Multicloning site; pH1, H1 promoter; pUb, ubiquitin promoter; SV40-poly A, polyadenylation sequence from Simian virus 40; T4, DNA ligase from bacteriophage, T4.

In order to assess the ability of the adenovirus to inhibit MCT1, different viral titers were tested on the HEK 293T cell line, which showed that 5 × 10^7^ IFU/mL was the most effective (nearly 100% transduction) and non-toxic titer. The adenoviral construction is shown in Fig. 1A. EGFP expression was used to monitor adenoviral transduction (Fig. 1B–G); high efficiencies were observed at 48 and 72 h (81.4% ± 7.4 for Adshβgal and 78.4% ± 6.8 for AdshMCT1 at 48 h; 78.8% ± 5.7 for Adshβgal and 84.6% ± 4.8 for AdshMCT1 at 72 h) with the highest infection rate after 96 h (88.9% ± 3.3 for Adshβgal and 91.7% ± 6.3 for AdshMCT1; Fig. 1H). Cell survival was also measured after adenovirus infection, which showed >90% of living cells at all times analyzed (Fig. 1I). MCT1 mRNA expression was quantified after 48, 72 and 96 h post-transduction by quantitative real-time polymerase chain reaction (Q-RT-PCR), and a significant reduction in MCT1 expression levels was detected at all times in the knockdown cultures, relative to cyclophilin and compared to control cultures (Fig. 1J). The effect of AdshMCT1 on MCT1 protein expression was also evaluated in total protein extracts of tanycyte cultures at 96 h post-transduction using EGFP as a transduction control and actin as a loading control (Fig. 1K). A significant decrease on MCT1 band intensity was detected, revealing an 80 ± 7.4% inhibition when compared with Adshβgal (Fig. 1L).

Additionally, we assessed MCT1 inhibition *in vitro* until 144 h (Supplementary Fig. S1). We detected EGFP fluorescence at 144 h post-transduction (Fig. S1A,B), and Western blot assays confirmed MCT1 inhibition from 48 to 144 h (Fig. S1C).

### *In vivo* MCT1 inhibition by adenoviral injection into the 3 V

**Figure 2:**
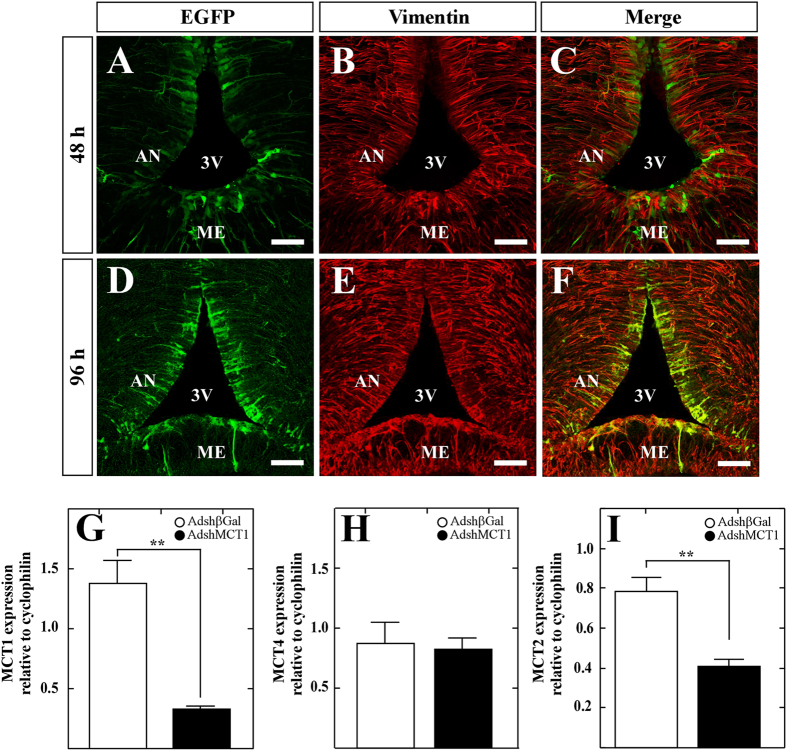
Adenoviral transduction and MCT mRNA expression in the hypothalamus of rats following icv injection in the 3V with AdshMCT1. (**A–F**) Frontal sections of the hypothalamus (40 μm) in which the immunoreactivity for vimentin (red) and for EGFP fluorescence (green) is shown in cells transduced with AdshMCT1 at 48 h (**A–C**) and 96 h (**D–F**) post-injection. (**G–I**) Q-RT-PCR analysis of MCT1 (**G**) MCT4 (**H**) and MCT2 (**I**) after 96 h of icv injection of AdshβGal (open bars) or AdshMCT1 (closed bars). AN, arcuate nucleus; 3V, third ventricle; ME, median eminence; *p < 0.05, **<0.01 (unpaired t-test). Scale bar: 150 μm.

Adenoviral particles carrying MCT1 or βgal shRNA were injected into the 3V of rats to evaluate the effect of MCT1 knockdown *in vivo*. Frontal brain sections of transduced animals were analyzed through confocal microscopy to detect EGFP fluorescence combined with vimentin immunostaining. EGFP expression was detected at 48 h post-transduction within tanycyte soma and processes (Fig. 2A–C); however, the largest number of EGFP-positive cells was detected at 96 h post-injection (Fig. 2D–F). Therefore, we chose 96 h after transduction for subsequent *in vivo* knockdown analysis.

We also evaluated MCT1 expression through Q-RT-PCR analysis of hypothalamic samples from rats injected with Adshβgal or AdshMCT1. MCT1 expression was significantly reduced by 76 ± 1.9% in animals receiving MCT1 shRNA compared with controls at 96 h post-injection (Fig. 2G). We also evaluated whether MCT4 expression, which is also present in tanycytes, was altered by MCT1 inhibition. We detected no significant differences in mRNA levels between both animal groups (Fig. 2H), demonstrating the specificity of the MCT1 shRNA. Moreover, we analyzed if the expression of MCT2, a transporter previously detected in hypothalamic orexigenic and anorexigenic neurons^9^, was disrupted by MCT1 inhibition given that MCT2 expression is altered in metabolic disorders^20^. MCT2 mRNA levels were decreased by 47.9 ± 5% in animals treated with AdshMCT1 compared to controls (Fig. 2I), suggesting that MCT1 inhibition in tanycytes does change MCT2 expression by hypothalamic neurons.

### Adenoviral injection into the 3V preferentially inhibits MCT1 expression by tanycytes

**Figure 3:**
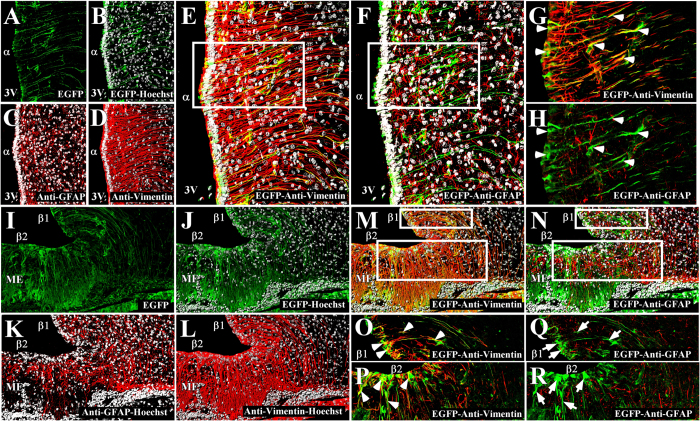
Co-distribution of EGFP and glial markers in the basal hypothalamus. (**A–R**) Rat frontal sections of the hypothalamus (40 μm) were analyzed using anti-vimentin (red) and anti-GFAP (red) antibodies as well as EGFP fluorescence (green) in cells transduced with AdshMCT1 at 96 h post-injection and the Hoechst nuclear stain (white). (**A–D)** Low magnification for EGFP (**A,B**) green, GFAP (**C**) red and vimentin (**D**) red expression in α-tanycytes. (**E,F)** 3D reconstruction of EGFP co-distribution with vimentin (**E**) and GFAP (**F**) in α-tanycytes. (**G,H)** One focal plane of the high magnification images of α-tanycytes for EGFP co-distribution with anti-vimentin (**G**) and anti-GFAP (**H**) antibodies. EGFP expression is observed in cells lining the 3V, co-localizing with vimentin (**G**) arrowheads and is not detected in GFAP-positive cells (**H**) arrows. (**I–L)** EGFP expression in β-tanycytes (**I–J**) green, GFAP (**K**) red and vimentin (**L**) red. (**M,N**) 3D reconstruction of EGFP co-distribution with vimentin (**M**) and GFAP (**N**) in β-tanycytes. (**O,P**) One focal plane of the high magnification images of β1-tanycytes (**O**) and β2-tanycytes (**P**) for EGFP co-distribution with vimentin. (**Q,R**) One focal plane of the high magnification images of β1-tanycytes (**Q**) and β2-tanycytes (**R**) for EGFP co-distribution with anti-GFAP. EGFP was expressed in both β1 and β2-tanycytes, co-localizing with vimentin (**O,P**) arrowheads and not with GFAP-positive cells (**Q,R**) arrows. α, α-tanycytes; β1, β1-tanycytes; β2, β-tanycytes; 3V, third ventricle; ME, median eminence. Scale bar: 150 μm.

**Figure 4:**
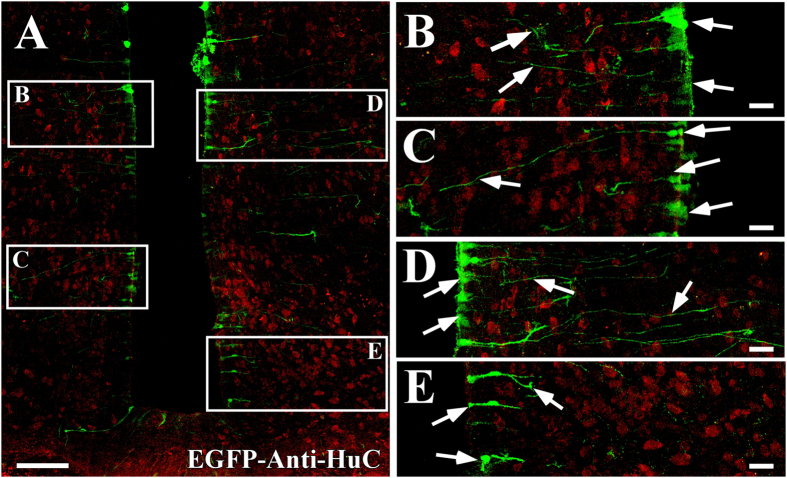
Co-distribution of EGFP and the neuronal marker, HuC, in the basal hypothalamus. (**A–E)** Frontal sections of the hypothalamus (40 μm) in which the immunoreactivity for Hu antibody (red) and for EGFP fluorescence (green) is shown in cells transduced with AdshMCT1 at 96 h (**D–F**) post-injection. (**A**) Low magnification showing that EGFP is detected in the ventricle wall and in the processes of tanycytes. (**B–E**) High magnification image of the frames shown in A. Scale bar: 150 μm.

It has been demonstrated that astrocytes generate lactate that can then be used by neighboring neurons as an alternative energy source, and this is especially important in situations of low glucose availability^21^,^22^,^23^,^24^. Adenoviral particles carrying MCT1 shRNA and the *EGFP* reporter gene were injected into the 3V of rats to evaluate if other glial cells were infected besides tanycytes at 96 h post-transduction. Frontal sections of the basal hypothalamus from transduced animals were analyzed by immunofluorescence and spectral confocal microscopy to detect EGFP (green), anti-GFAP (red), anti-vimentin (red), and the nuclear marker, Hoechst (white) (Fig. 3A–R). EGFP expression was detected in ventricular cells with elongated processes, which due to its location, corresponds to α-tanycytes (Fig. 3A–H). A three dimensional (3D) reconstruction of this region showed that the EGFP-positive cells were negative for GFAP and positive for vimentin (Fig. 3E,F), which is best observed at high magnification using one focal plane (Fig. 3G,H). We also detected EGFP in the cell bodies and processes of cells lining the infundibular recess, which according their location, correspond to β1-tanycytes (Fig. 3I,J). Also, EGFP was detected in cells that cover the base of the 3V and contact the ME, corresponding to β2-tanycytes (Fig. 3I,J). In both cases, EGFP exhibited a clear co-localization with vimentin (Fig. 3M), which was most evident at higher magnification (Fig. 3O,P, arrowheads). GFAP was also detected in this area (Fig. 3N,K, red), but its distribution did not correlate with vimentin (Fig. 3M,L, red), which is in agreement with previous results^8^. GFAP was primarily detected in the subependymal region of the ME and in the processes of dorsal β1-tanycytes as was previously described^8^. EGFP fluorescence was not detected in astrocytes located in the lateral AN (Fig. 3Q, arrows) or in the ME (Fig. 3R, arrows). We also evaluated EGFP fluorescence in AN neurons using a HuC antibody, a neuronal marker (Fig. 4, red). Low magnification images (Fig. 4A) showed no evident EGFP expression (Fig. 4, green) in the neuronal bodies, which is better appreciated in the high magnification images (Fig. 4B–D). EGFP fluorescence was detected only in cells with a tanycytic phenotype (Fig. 4B–E, arrows). These results indicate that the adenovirus preferentially transduced tanycytes.

### Neuropeptide expression in response to icv glucose in rats following MCT1 knockdown

**Figure 5:**
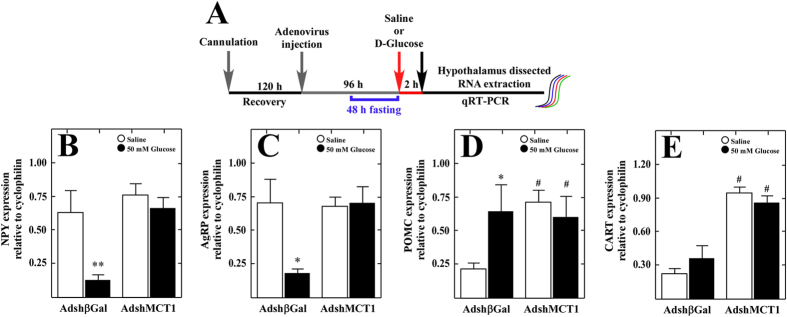
MCT1 inhibition impairs both orexigenic and anorexigenic neuropeptide expression. (**A**) Scheme of the experimental protocol. Adult male rats were sterotaxically cannulated into the 3V. After 96 h of recovery, the rats were injected with AdshβGal or AdshMCT1. At 48 h post-injection, the rats were fasted for 48 h and subsequently injected with saline buffer or 50 mM D-glucose. At 2 h post-3V icv injection, the animals were sacrificed, and the hypothalamus was dissected for RNA extraction and Q-RT-PCR analysis. (**B)** Q-RT-PCR analysis of NPY, AgRP, POMC and CART neuropeptide expression in rats transduced with AdshβGal for 96 h. (**C,D**) Q-RT-PCR analysis of NPY/AgRP (**C**) and POMC/CART (**D**) neuropeptide expression in rats transduced with AdshMCT1 relative to AdshβGal neuropeptide expression. *^,#^*p* < 0.05, **^,##^*p* < 0.01 (t-test, followed by a Mann-Whitney U-test).

It has been previously shown that a 50 mM glucose stimulus directly into the 3V generates a neuronal response mediated by changes in anorexigenic and orexigenic neuropeptide expression^25^. Therefore, we tested if this glucose response was maintained in MCT1 knockdown rats transduced for 96 h and fasted for 48 h (Fig. 5A). A total of six rats were analyzed per condition. Neuropeptide expression was measured by Q-RT-PCR after 2 h of saline or icv D-glucose (Fig. 5B–E). After glucose injection, control Adshβgal rats had a 4-fold reduction in the expression of the orexigenic neuropeptides, NPY (Fig. 5B, closed bar) and AgRP (Fig. 5C, closed bar), and a 3-fold increase in the expression of the anorexigenic neuropeptide, POMC (Fig. 5D, closed bar), relative to rats treated with saline (Fig. 5B–E, open bars). We also observed an increased expression of CART after the glucose stimulus (Fig. 5E, closed bar); however, it was not statistically significant compared with saline treatment (Fig. 5E, open bar). These results agree with those reported by Bady *et al.*^25^ which showed the expected normal response to glucose.

We next evaluated the effect of MCT1 inhibition on orexigenic and anorexigenic neuropeptide levels. MCT1 inhibition produced a loss of response to icv glucose injection, showing expression levels of NPY (Fig. 5B, closed bar) and AgRP (Fig. 5C, closed bar) similar to those observed with the saline treatment (Fig. 5B,C, open bars). In contrast, the anorexigenic neuropeptides, POMC (Fig. 5D, open bar) and CART (Fig. 5E, open bar), showed altered expression levels under saline treatment (fasting conditions), exhibiting 3 to 4-fold higher levels than AdshβGal control animals (Fig. 5D,E, open bars). We did not detect changes in anorexigenic neuropeptide expression after icv glucose injection (Fig. 5D,E, closed bars), compared with the basal levels observed following saline treatment (Fig. 5D,E, open bars). Taken together, our results show that MCT1 inhibition impairs both orexigenic and anorexigenic neuropeptide responsiveness to increased glycorrhachia, possibly resulting in hunger/satiety signal dysregulation. To test these assumptions, we evaluated the impact of MCT1 inhibition on body weight and eating behavior.

### Feeding behavior of rats following MCT1 inhibition

After cannulation, feeding behavior was monitored and compared to non-cannulated animals. During the first three days after the cannula implantation, the food intake of cannulated animals was significantly less than controls. However, at day 4, normal feeding behavior was observed (Fig. S1). For this reason, we administered the adenoviruses on day 5 (120 h) post-cannulation.

**Figure 6:**
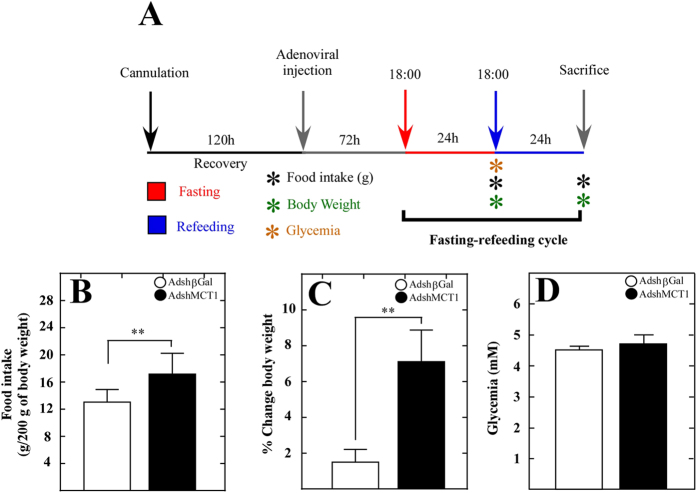
Knockdown of MCT1 increases food intake and leads to a sustained intake over time. (**A**) Scheme of the experimental protocol. Adult male rats were sterotaxically cannulated into the 3V. After 120 h of recovery, the rats were injected with AdshβGal or AdshMCT1. At 72 h post-adenoviral injection, the rats were subjected to 2  h of fasting followed by 24 h of feeding. Parameters, including food intake, body weight, glycemia and meal frequency, were measured. (**B**) Quantification of food intake by rats transduced with AdshβGal (open bars) or AdshMCT1 (closed bars) over 24 h after feeding were expressed as g/200 g body weight. (**C)** Percentage of change in body weight 24 h after feeding in rats transduced with AdshβGal (open bars) or AdshMCT1 (closed bars). (**D)** Quantification of glycemia after fasting in rats transduced with AdshβGal (open bars) or AdshMCT1 (closed bars). **p < 0.01 (*unpaired t-test).

After 72 h of MCT1 shRNA or β-gal shRNA adenoviral injection, the rats were subjected to a fasting-feeding cycle that consisted of 24 h of fasting followed by 24 h of feeding. After each period of time, food intake, body weight and glycemia were measured (Fig. 6A). A total of 10 rats per condition was analyzed. MCT1 inhibition resulted in a significant increase in food intake by 4.5 g as compared to control rats (Fig. 6B). Body weight was also increased significantly by 5.5 g as compared to control rats (Fig. 6C). In order to demonstrate that the changes detected in food intake and body weight were not due systemic glycemic alterations, blood glucose concentrations were measured. No significant difference was found between the control and shMCT1 groups (Fig. 6D). These results could be explained, at least in part, as an altered communication between tanycytes and neurons, due to MCT1 inhibition. However, we cannot discard that at over 96 h post adenovirus injection, a compensatory response by neurons or peripheral signals could also explain the results described above.

**Figure 7:**
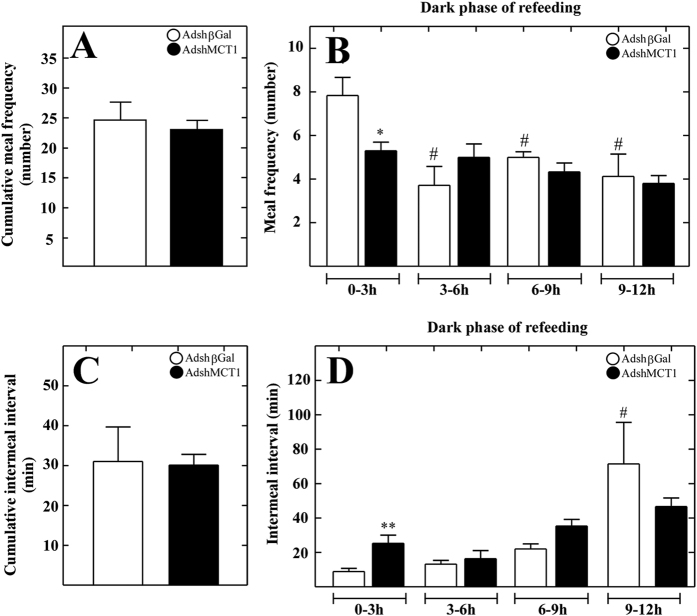
MCT1 knockdown alters the satiety behavior. (**A–D**) Rats were transduced with AdshβGal (open bars) or AdshMCT1 (closed bars). (**A**) Cumulative meal frequency over 24 h was determined. **(B)** Detailed analysis of meal frequency every 3 h in the dark phase. In controls, the meal frequency decreased while remaining relatively constant in shMCT1 animals. (**C**) Cumulative intermeal-intervals over 24 h were determined. (**D**) Quantification of intermeal-intervals every 3 h in the dark phase. In the controls, the duration of the intervals increased while remaining relatively constant in shMCT1 animals, suggesting that MCT1 knockdown disrupted satiety signaling. ANOVA: ^#^*p* < 0.05, **p < 0.01 (*unpaired t-test), (^#^paired t-test).

A general analysis of cumulative meal frequency, defined as the number of times that a feeding event occurs, showed no differences compared with the control group (Fig. 7A). However, a more detailed analysis focusing on the meal frequency in several periods of time during the dark phase revealed that during the first 3 h, shMCT1 rats showed a significantly lower meal frequency than the control group (Fig. 7B). During the second period (3–6 h), the control group experienced a 50% reduction, which was maintained in the following periods of time: 6–9 h and 9–12 h. However, shMCT1 rats showed no such reduction. Specifically, no differences were detected when was compared with those obtained in the first period (0–3 h) or with control rats of the same period (Fig. 7B). In addition, no differences were noticed in the two subsequent periods of time (Fig. 7B, 6–9 h and 9–12 h). The feeding behavior of shMCT1 rats differed from control rats in that i) they had a lower frequency of feeding immediately after fasting, which can be interpreted that these animals were less hungry and ii) they did not experience a drop in intake after the first 3 h, which could be attributed to a delay in establishing satiety. Because these results suggest an inhibitory effect on satiety, we analyzed the inter-meal intervals in the same periods of the dark phase, as this parameter also reflects satiety behavior. A general analysis of inter-meal intervals showed no differences compared with the control group (Fig. 7C). However, a more detailed analysis revealed that during the first 3 h, shMCT1 rats showed a significantly higher inter-meal interval than observed for the control group (Fig. 7D). These results were in agreement with the observed decrease in meal frequency during the first 3 h (Fig. 7B). Because meal frequency and inter-meal interval are both considered satiety indicators, our results suggest that MCT1 inhibition impairs the mechanisms that cause a delay in the initiation of a new meal, reducing the meal frequency in the first 3 h of the cycle. In the last period (9–12 h), animals injected with Adshβ-Gal had a three-fold increase in inter-meal intervals compared to the previous period. However, in AdshMCT1-injected animals, the inter-meal intervals remained constant, suggesting that MCT1 inhibition reduced the satiety in this interval of time (Fig. 7D). Using these data, other parameters of feeding behavior, including eating rate, meal duration and meal size, were calculated as mean values for the whole cycle without making a distinction between phases (Table 1). Analysis of the eating rate, estimated as the total amount of food consumed in total meal duration, showed that shMCT1 rats had a higher eating rate, which correlated with the highly significant increase in food intake (Fig. 6B). No differences between the shMCT1 and control rats were detected for the satiation indicators, mean meal duration and mean meal size (Table 1).

## Discussion

Glia-neuron coupling regulates important physiological and pathophysiological events in different brain regions, including the cortex, cerebellum and hippocampus. These events include synaptogenesis^27^, brain stroke, long-term memory formation and metabolic functions (e.g., glutamate-glutamine recycling, ammonium fixation, glycogen storage, maintenance of neuronal energy metabolism and detoxification of free radicals)^24^,^28^. Recent evidence suggests that the hypothalamic glucosensing mechanism is also an example of indirect interaction through a metabolic coupling between glia and neurons^11^,^12^,^15^. Moreover, we have previously demonstrated the expression of several proteins involved in detecting and responding to glucose in tanycytes; however, there was no evidence of their involvement in the regulation of food intake^2^,^29^,^30^,^31^,^32^. Here, we generated an efficient molecular tool for knocking down MCT1 gene expression *in vitro* and *in vivo*. shMCT1 animals had an evident loss of response to icv glucose as detected by altered neuropeptide expression, and a mild alteration in feeding behavior. These data support a role for MCT1 in hypothalamic glucosensing.

Our *in vitro* studies show that MCT1 expression was significantly decreased at 96 h post-transduction. Although we did not detect differences in EGFP expression at the different times evaluated in our *in vitro* analysis, we did observe changes between 48 and 96 h *in vivo*. According to the localization and morphological features of the EGFP-positive cells observed *in vivo* along with vimentin, GFAP and HuC expression, our results suggest that the adenoviral particles preferentially transduce tanycytes.

Immunolocalization studies have shown that MCTs have a differential distribution in the basal hypothalamus with MCT1 and MCT4 located on tanycyte processes^8^, and MCT2 located on orexigenic and anorexigenic neurons of the AN^8^,^33^. MCT1 shRNA produces a significant decrease in MCT1 mRNA expression without altering MCT4 mRNA expression; however, a partial reduction in MCT2 mRNA levels was detected. This could not be explained by low specificity of the shRNA used because it should not recognize MCT2 as a target according to the MCT2 sequence. In this regard, it has been demonstrated that MCT2 expression is upregulated by increases in extracellular monocarboxylate concentrations^34^, as has been reported in neonatal brains, whose high levels of ketone bodies from hepatic oxidation of milk lipids produce higher levels of expression than in adult stages^35^,^36^. Therefore, it is possible that inhibition of MCT1 decreases monocarboxylates release, which could lead to a reduction in MCT2 expression. Further studies will be necessary to evaluate the function of MCT2 and MCT4 on food intake.

Hypothalamic neuronal circuits play an important integrative role in the control of feeding and are regulated by internal hormonal and nutrient signaling^6^,^37^,^38^,^39^,^40^. These signals impact AN neurons, regulating the expression and release of hypothalamic orexigenic and anorexigenic neuropeptides^15^,^41^,^42^,^43^. It has been demonstrated that during fasting, NPY and AgRP expression is upregulated, while after icv glucose injection, the levels of POMC and CART increased^25^,^44^. Our results with control rats (treated with AdshβGal) showed the same pattern. In contrast, the shMCT1 animals had elevated levels of orexigenic neuropeptides after glucose injection compared with the saline control, which may suggest that they were not directly inhibited by glucose. It has been postulated that orexigenic neurons are inhibited by tanycytic lactate, which is released in response to increased glucose^4^,^8^,^45^. In MCT1 knockdown rats, inactivation of these neurons in response to glucose would not occur, which may explain why orexigenic neuropeptide levels are similar to the saline control levels. Similar results have been obtained in a ripglut1;glut2^−/−^ mice, in which icv injection of the same glucose concentration did not decrease orexigenic neuropeptide expression^25^. In this context, we have previously shown that tanycytes express GLUT2 and respond to increases in glucose concentration, supporting the participation of tanycytes in controlling food intake^2^,^30^. Additionally, a partial reduction in MCT2 mRNA levels could be responsible for part of the observed effects following MCT1 inhibition *in vivo*, reinforcing the MCT1 knockdown effect. Nevertheless, our results support a metabolic coupling between tanycytes and neurons.

Several studies support that AN neurons could respond to glucose; those performed in brain slices showed that 40% of NPY neurons are inhibited by glucose^41^,^46^,^47^,^48^,^49^,^50^. Similarly, a population of glucose-excited neurons, which depolarize and increase their firing rate in response to increases in extracellular glucose via the closure ATP-sensitive potassium (K_(ATP)_) channels, has been identified^40^. Furthermore, disruption of glucosensing in glucose-excited POMC neurons via transgenic expression of a mutant Kir6.2 subunit that prevents closure of K_(ATP)_ suppresses the response to a systemic glucose load, demonstrating a role for POMC neurons in the overall physiological control of blood glucose^17^. These clear demonstrations of neuronal involvement in hypothalamic glucosensing mechanism are not in contradiction with our results, because both glucose as well as lactate could generate the response mediated by ATP in these neurons. Since AN neurons are not in direct contact with blood or CSF^4^,^51^,^52^, and tanycytes are in contact with both, it is possible that an alternative or parallel pathway is supporting this process. Surprisingly, anorexigenic neuropeptide expression was also altered in fasting after MCT1 inhibition. It is possible that other substrates transported by MCT1 decrease the activity of these neurons in this condition. MCTs are a family of transporters, which mediate facilitated diffusion of lactate and several other metabolically important monocarboxylates, such as pyruvate and ketone bodies^8^,^10^,^53^. It has been demonstrated that ketone body levels are increased with fasting^10^, so it is possible that the monocarboxylates released by tanycytes could contribute to this neuronal response. Supporting this notion, numerous lipid droplets and the expression of enzymes involved in lipid metabolism have been histochemically demonstrated in tanycytes^54^. Furthermore, it has been shown that cultured astrocytes could release ketone bodies that are generated by the catabolism of fatty acids^55^,^56^. However, it cannot be excluded that a reduction of MCT1 expression in tanycytes may affect the uptake of ketone bodies synthesized in the liver by the brain. Also, the immunocytochemical data presented in the present study show that the adenovirus preferentially transduced vimentin-positive cells with classical tanycyte morphology. Nevertheless, endothelial cells are also vimentin-positive; therefore, further studies are required to demonstrate the relative contribution of both mechanisms. Interestingly, the expression of POMC and CART was similar to the saline control group, which may be due to a partial loss of response to glucose that is expected since these neurons are in close contact with tanycyte processes that are mostly positive for MCT4 and not MCT1^8^.

It has been demonstrated that icv injection of 5 mM lactate leads to a 58% reduction in food intake and body weight in rats^15^,^26^. Our data support the hypothesis that tanycyte-neuron coupling mediated by monocarboxylates has a fundamental role in food intake since shMCT1 animals had higher food intake and body weight. However, food intake is a behavior regulated by several factors, including nutrient availability, which may explain why we did not detect differences in cumulative frequency or inter-meal intervals (12 h dark/12 h light). However, frequency analysis at shorter intervals allowed us to detect alterations in the feeding behavior. During the first 3 h of the dark phase, knockdown animals had decreased meal frequency, which correlated with longer wait times between meals. However, our results showed that during the whole cycle, shMCT1 rats eat more and faster than control rats. This suggests that shMCT1 animals may have reduced hunger sensations in the first post-fasting hours than control animals, which could be due the high levels of anorexigenic neuropeptides found in the fasting condition. Moreover, it has been recently demonstrated that liver-derived ketone bodies are necessary for food anticipation, signaling presumably through hypothalamic nuclei^57^. This mechanism can also be affected by MCT1 inhibition; therefore, the delay in feeding initiation observed in shMCT1-treated animals can be attributed in part to a disruption in ketone body uptake. However, after 6 h, the food frequency in shMCT1 animals was stabilized, which is in contrast to the significant drop in frequency observed in the control group. Over the study period, control animals showed an increase in meal frequency and a reduction in intervals between meals. In contrast, both parameters remained constant in shMCT1 animals in all of the time periods analyzed. The large food intake could be explained by the fact that shMCT1-treated animals have a higher feeding rate with respect to the control group. Additionally, if the neuropeptide expression levels are maintained, as we detected at 96 h post injection, the high food intake could be due in part to elevated orexigenic neuropeptide levels. However, because our feeding cages are not able to detect variations in food weight at each meal, this parameter was measured at the end of the cycle; therefore, possible increases in the parameters, which measure satiation, may have been undetected.

The delays in food intake initiation and satiety establishment show that hypothalamic signaling mediated by MCT1 is relevant under low- and high-glucose conditions, and it is needed to maintain normal food intake. In addition, it has recently been shown that MCT1^+/−^ mice fed a high-fat diet exhibit resistance to obesity, as well a reduced insulin resistance and decreased hepatic steatosis^58^. Interestingly, MCT1^+/−^ animals have normal blood lactate and ketone body levels, but reduced insulin and leptin levels. These findings uncover the important role of MCT1 in the regulation of energy balance in animals exposed to an obesogenic diet^58^ and further implicate a role for MCT1 in feeding behavior. Moreover, it has been demonstrated that lactate transport and metabolism controls plasma glucose and lipid levels^15^,^26^. Therefore, new therapeutic strategies involving the modulation of monocarboxylate transporters in the CNS could be used to control obesity and its associated pathologies.

## Materials and Methods

### Ethics Statement

All animal experiments were in accordance with the Guidance on the Operation of the Animals (Scientific Procedures) Act 1986, and all animal studies were approved by the appropriate Ethics and Animal Care and Use Committee of the Universidad de Concepcion, Chile (permit number 2010101A). Male adult Sprague-Dawley rats weighing 250–300 g were used in all experiments. Animals were housed in a separate animal room with constant temperature (21 ± 2 °C) and a controlled 12-h light/ 12-h dark cycle; lights were turned on every day at 7:00 a.m. Animals had free access to a standard rodent diet (Lab Diet, 5P00 Prolab RMH 3000, Purina Mills, St. Louis, MO) and tap water.

### Preparations of Adenoviral shRNA-MCT1 Vectors

The sequence targeting rat MCT1 (GenBank: D63834.1) was selected using siDESIGN Center (Dharmacon RNAi technologies), and sequences with homology with other rat coding sequences by BLAST analysis were discarded. The following oligonucleotides were used: sense 5′-CGC GCC GCA GCT TCT TTC TGT AAC ATT CAA GAG ATG TTA CAG AAA GAA GCT GCT TTT TTT TAA T-3′ and antisense 5′-TAA AAA AAA GCA GCT TCT TTC TGT AAC ATC TCT TGA ATG TTA CAG AAA GAA GCT GCG G-3′. A ring sequence of nine base pairs (TTC AAG AGA) existed between the sense and antisense strands. Control siRNA oligonucleotides were designed and selected to target β-galactosidase from *E*. *coli:* sense 5′-CGC GCC AAG GCC AGA CGC GAA TTA TTT CAA GAG AAT AAT TCG CGT CTG GCC TTT TTT TTT TAA T-3′ and antisense 5′- TAA AAA AAA AAG GCC AGA CGC GAA TTA TTC TCT TGA AAT AAT TCG CGT CTG GCC TTG G-3′. Cloning of the expression cassette into the adenoviral shuttle vector was then performed. Briefly, the EcoRI and SacI fragment encoding the H1-promotor, multicloning site (MCS), ubiquitin promoter, EGFP and SV40 polyA from the Fux vector^59^ was ligated into the EcoRI and SacI sites of the pDC311 adenoviral shuttle expression vector (Microbix, Ontario, Canada). The shRNA was cloned into the MCS through the AscI and PacI sites. The adenoviral expression system was produced in HEK293A cells by cotransfecting cells with pBHGlox(Δ)E1,3Cre (Admax system, Microbix biosystems Inc. Ontario, Canada) adenoviral genomic DNA and either pDC311-H1-shMCT1-Ub-EGFP or the pDC311-H1-shbGal-Ub-EGFP expression vectors. The resulting adenoviral expression vectors were titered by EGFP expression using the Adeno-XTM Rapid Titer Kit Protocol (Clontech). After amplification, adenoviral particles were purified using the VirakitAdenoMini-4 kit (Virapur, USA), aliquoted and stored at −80 °C.

### Primary culture of tanycytes

Hypothalamic tanycyte cultures from 1-day postnatal brains were prepared following the method described previously^2^,^8^,^30^,^32^. Briefly, the hypothalamic region was removed from the brain and further dissected to obtain the tissue containing the ependymal layer. Trypsinized tissue was transferred to tissue culture plates containing MEM medium, (Invitrogen, Carlsbad, CA, USA) with 10% (v/v) fetal bovine serum (FBS) (Thermo Fisher Scientific Inc., Waltham, MA, USA) and 2 mg/mL DNase I (Sigma-Aldrich, St. Louis, MO, USA). Dishes with the highest density of confluent epithelial cells were expanded for subsequent adenoviral transduction to measure cell survival, transduction efficiency and protein expression.

### Adenoviral transduction *in vitro*

To measure cell survival and transduction efficiency, cells were grown on poly-L-lysine-coated glass cover slides in 24-well plates in MEM medium supplemented with 10% (v/v) FBS. Cells were transduced with Ad-MCT1-shRNA or Ad-βGal-shRNA (control) at 5 × 10^7^ infectious units per mL (IFU/mL). Virus-containing medium was replaced 24 h later with MEM medium containing 10% (v/v) FBS and incubated for a total of 48, 72 and 96 h. Survival was measured by the trypan blue exclusion assay. Transduction efficiency was calculated as the percentage of total cells obtained using the nuclear marker, TOPRO-3 (1:1000, Invitrogen), and also EGFP-positive. Coverslips were visualized by confocal microscopy LSM 700 (Zeiss, Germany) after fixation with 4% paraformaldehyde (PFA).

### Cannula implantation

Cannulae were stereotaxically implanted into the 3V with the following protocol. Rats were anesthetized with an intraperitoneal injection mix of ketamine-xilazine (90 mg/kg–10 mg/kg), and the fur at the top of the head was removed to expose the area to be incised. A hole was drilled in the skull, and a guide cannula (28 gauge stainless steel; Plastics One, Roanoke, VA) was lowered using the following stereotaxic coordinates: anterior-posterior from bregma −3.14 mm, medial-lateral from midsaggital sinus 0.0, and dorsal-ventral from the top of the skull 9.2 mm. The guide cannula was secured to the skull using 3/32 mm mounting screws and dental acrylic. A removable dummy cannula (28 gauge stainless steel; Plastics One, Roanoke, VA) fit into the cannula guide, sealing the opening in the guide cannula throughout the experiments except when it was removed for the injections. Rats were housed individually following surgery and allowed to recover for 5 days before adenovirus administration and starting the experimental procedures.

### icv injections of shMCT1 and shβGal adenovirus

Rats were anesthetized with isoflurane and then injected into the 3V with 30 μL of 2 × 10^9^ IFU/mL (2.5 μL/min). For the mRNA expression analysis, rats were injected with adenovirus as described in Fig. 4A. Subsequently, the rats were anesthetized with isoflurane and injected with 10 μL of saline buffer (128 mM NaCl, 3 mM KCl, 1.3 mM CaCl_2_, 1.0 mM MgCl_2_, 1.3 mM NaH_2_PO_4_, 21 mM Na_2_HPO_4_, pH 7.4 and 320 mOsm) or 10 μL of 50 mM D-glucose diluted in the same buffer (320 mOsm, pH 7.4). Hypothalamic samples were collected after 2 h post-glucose or saline injection for the mRNA analysis expression. For the protein analysis, hypothalamic samples were collected after 96 h post-adenoviral injection. At 72 h post-adenoviral injection, rats were subjected to a 24-h fasting period followed by 24-h feeding period for the feeding behavior analysis.

### Measurement of hypothalamic mRNA

Q-RT-PCR analysis was used to measure the expression of the hypothalamic cyclophilin, MCT1, MCT2, MCT4, NPY, AgRP, POMC and CART. The following sets of primers were used: cyclophilin, sense 5′-ATA ATG GCA CTG GTG GCA AGT C-3′ and antisense 5′-ATT CCT GGA CCC AAA ACG CTC C-3′; MCT1, sense 5′-TGG AAT GTT GTC CTG TCC TCC TGG-3′ and antisense 5′-TCC TCC GCT TTC TGT TCT TTG GC-3′; MCT2, sense 5′-CTA TCG TGG AGT GTT GCC CAG TTC-3′ and antisense 5′-CAT TTC TTT GGA TGC CTG CGA G-3′; MCT4, sense 5′-TTC TCC AGT GCC ATT GGT CTC GTG-3′ and antisense 5′-CCC GCC AGG ATG AAC ACA TAC TTG-3′; NPY, sense 5′-TGT TTG GGC ATT CTG GCT GAG G-3′ and antisense 5′-CTG GGG GCA TTT TCT GTG CTT TC-3′; and AGRP, sense 5′-GCA GAC CGA GCA GAA GAT GTT C-3′ and antisense 5′-GTA GCA CGT CTT GAA GAA GCG G-3′; POMC, sense 5′-CTC CTG CTT CAG ACC TCC ATA GAC-3′ and antisense 5′-AAG GGC TGT TCA TCT CCG TTG-3′; and CART, sense 5′-TCT GGG AAG AAG AGG GAC TTT CGC-3′and antisense 5′-TCC ATT TGT GTT GCT TTG GGG TG-3′. First, the brain of each rat was removed, and the hypothalamic area (Bregma −1.74/−4.56) was isolated and further dissected to obtain a region close to the 3V ependymal layer. The reverse transcription was performed according to the manufacturer’s protocol of M-MULV reverse transcriptase (Fermentas International INC). PCR reactions were carried out in an Mx3000P QPCR System (Agilent Technologies, Santa Clara, CA, USA). Q-RT-PCR was performed using the qPCR Master Mix kit for Brilliant II SYBR Green (Agilent Technologies, Inc.) in a final volume of 12.5 μL consisting of 1x SYBR green Master Mix, 500 nM of each primer and 1 μL of cDNA sample. All reactions were performed with an initial denaturation of 5 min at 95 °C, followed by 40 cycles of 30 s at 95 °C, annealing for 30 s at 55 °C, and extension for 1 min at 72 °C. The relative expression of MCTs or neuropeptides to cyclophilin mRNA was calculated on the basis of the PCR efficiency.

### Immunoblotting

Total protein extracts were obtained from rat hypothalamic samples and primary cultures of tanycytes. Samples were homogenized in protease inhibitor cocktail (ROCHE) and sonicated three times on ice at 300W. Proteins were resolved by SDS-PAGE (50 μg/lane) in a 5–15% (w/v) polyacrylamide gel, transferred to PVDF membranes (0.45 μm pore, Amersham Pharmacia Biotech., Piscataway, NJ, USA), and probed for 2 h at 4 °C with a chicken anti-MCT1 (1:1000, MERCK, Darmstadt, Germany) antibody. After extensive washing, the PVDF membranes were incubated for 1 h at 4 °C with peroxidase-labeled rabbit anti-chicken IgY (1:1000; Jackson ImmunoResearch Laboratories, Inc., PA, USA). The reaction was developed using the enhanced chemiluminescence (ECL) Western blot analysis system (Amersham Biosciences).

### Immunocytochemistry

In order to analyze the specificity of the adenovirus *in vivo,* the animals were injected with the adenovirus, and brains were collected at 48 and 96 h. The rat brains were fixed in 4% PFA by immersion for 48 h. After fixation, thick frontal sections of the hypothalamus (40 μm) were cut with a cryostat, and subsequently free-floating processed. Tissues were stained with mouse anti-vimentin (1:200, DAKO), rabbit anti-GFAP (1:200, DAKO) and mouse anti-HuC (1:200, Invitrogen). The antibody was diluted in a Tris-PO_4_ buffer (pH 7.8) containing 1% bovine serum albumin. After extensive washing, the sections were incubated for 2 h at room temperature with Cy2- or Cy3-labeled secondary antibodies (1:200; Jackson ImmunoResearch Laboratories). These samples were counterstained with the DNA stain, TOPRO-3 (1:1000; Invitrogen). The slides were analyzed using confocal laser microscopy LSM 700 (Zeiss).

### Measurement of blood glucose

Blood samples for glucose measurements were taken by needle puncture from the tail vein after 24 h in a fasting condition to ensure a hypoglycemic state. Blood glucose measurements performed on whole blood were made with an Accu-Chek Go (Roche) glucometer.

### Measurement of food intake

Rats were handled for one week everyday to become accustomed to the researchers and experimental procedures. This included removal of the rat from the cage to measure food intake and body weight monitoring. Food intake was monitored by providing rats with pre-weighed rat chow, and food was weighed after a defined time interval. Food intake was expressed as g consumed per 200 g of body weight (g/200 g body weight). Every interaction with the feeder was recorded by a computerized data acquisition system (VitalView, Respironics, Inc, Murraysville, PA, USA).

A meal was considered when the bouts at the feeder were larger than 5 sec, and these meals were separated from other feeding bouts by more than 10 min of intermeal interval^10^,^14^. When bouts of feeding were longer than 30 min, they were considered two meals. The meal pattern parameters were calculated as follows: intermeal interval (min), meal frequency (number), cumulative meal frequency (number), mean meal size (mg/meal), mean meal duration (min/meal), and eating rate (mg/min). The inter-meal interval was calculated as the period between the end of one meal and the initiation of the next. The cumulative meal frequency was defined as the total meals in 24 h. The mean meal size was determined as the total food intake (mg) divided by frequency. The mean meal duration was calculated by dividing the total meal duration (min) by meal frequency, and the eating rate was estimated by dividing total food intake (mg) by total meal duration (min).

### Statistical analyses

Results were expressed as mean ± standard error of the mean (SEM), and n refers to the number of animals used. For statistical analysis, each treatment was compared with its respective control. Differences between two groups for evaluating qRT-PCR assays were assessed using the Student t-test, followed by a Mann-Whitney U-test using. Differences between groups in feeding behavior assays were assessed using ANOVA. Differences were considered significant when *P* < 0.05. The statistical analyses were performed using GraphPad Prism 5.0 Software (GraphPad Software Inc., San Diego CA, USA).

## Additional Information

**How to cite this article**: Elizondo-Vega, R. *et al.* Inhibition of hypothalamic MCT1 expression increases food intake and alters orexigenic and anorexigenic neuropeptide expression. *Sci. Rep.* **6**, 33606; doi: 10.1038/srep33606 (2016).

## Supplementary Material

Supplementary Information

## Figures and Tables

**Table 1:** Microstructure of the ingestion in MCT1 knockdown animals.

Parameter	AdshßGal	SD	AdshMCT1	SD
Eating rate (mg/min)	57.6	8.1	68.3*	9.0
Mean meal duration (min)	11.7	2.3	12.0	2.0
Mean meal size (mg)	0.76	0.2	0.78	0.18

*p < 0.01 t-test.
