# Supplementary material for: Inhibition of hypothalamic MCT1 expression increases food intake and alters orexigenic and anorexigenic neuropeptide expression
Source: Sci Rep. 2016 Sep 28;6:33606. doi: 10.1038/srep33606 (PMC5039692; doi:10.1038/srep33606)
Supplement: Supplementary Information [file srep33606-s1.doc]

**Inhibition of hypothalamic MCT1 expression increases food intake and alters orexigenic and anorexigenic neuropeptide expression**

Elizondo-Vega Roberto^1&^, Cortés-Campos Christian^1,2&^, Barahona María José^1^, Carril Claudio^1^, Patricio Ordenes^1^, Salgado Magdiel^1^, Oyarce Karina^1,3^, García-Robles María Angeles^1*^

^1^ Departamento de Biología Celular, Facultad de Ciencias Biológicas, Universidad de Concepción, Concepción, Chile, ^2^ Whitehead Institute for Biomedical Research, Cambridge, Massachusets, 02142, USA, ^3^Facultad de Medicina, Universidad San Sebastián, Concepción, Chile.

^&^Equal contribution


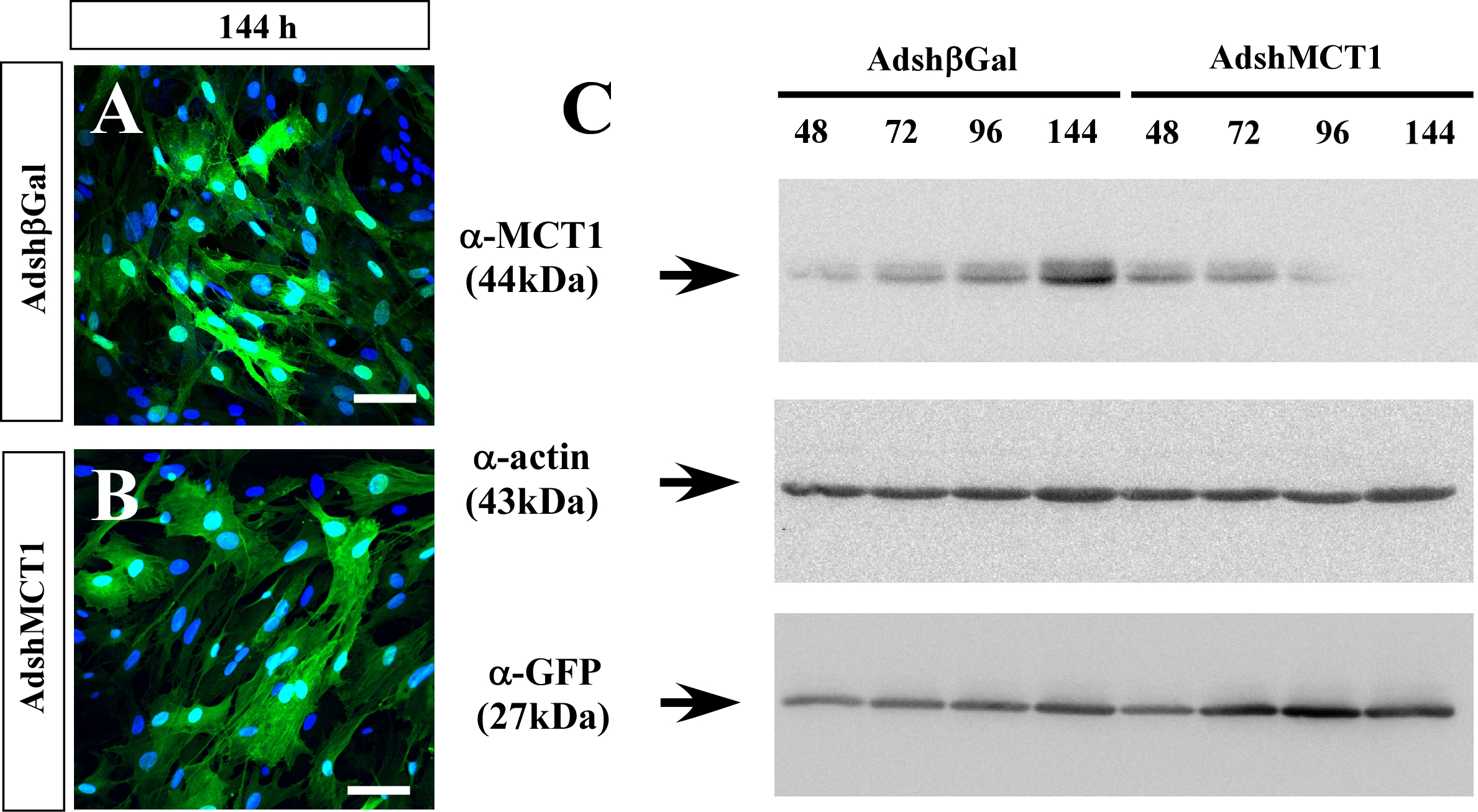


**Figure S1: MCT1 inhibition in cultured tanycytes for up to 144 h.**

**A-B:** Temporal EGFP expression in tanycytes cultures transduced for 144 h with AdshβGal (A) or AdshMCT1 (B). Nuclei were stained with TOPRO-3 (blue). Western blot analysis of MCT1, GFP and actin. Lanes 1-4: Total cell extracts at 48, 72, 96 and 144 h post-transduction with AdshβGal; lanes 5-8: total cell extracts at 48, 72, 96 and 144 h post-transduction with AdshMCT1.


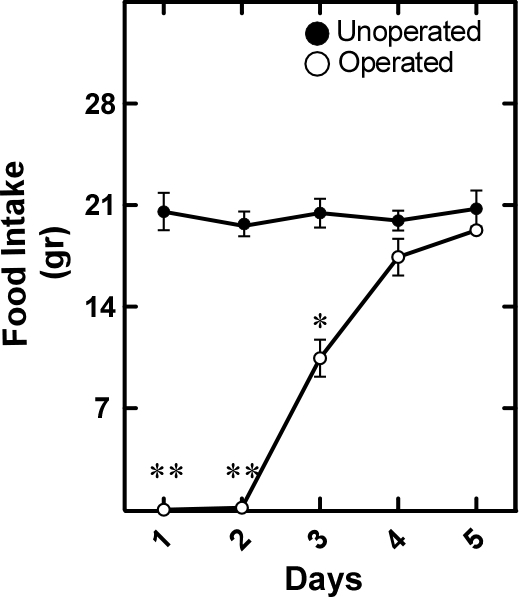


**Figure S2: Food intake recovery of cannulated animals prior to injection of adenovirus.**

Grams of food consumed by animals over the 5 days after cannulation were measured and compared to unoperated control rats. Feeding pattern in cannulated animals was restored by day 4; therefore, the rats were injected with adenovirus on day 5 post-cannulation. N: 10 * *p*<0.05, ***p*<0.01 (unpaired t-test).
